# Bullet Embolization After a Penetrating Gunshot Wound: A Case Report

**DOI:** 10.7759/cureus.44261

**Published:** 2023-08-28

**Authors:** Elen Mussie, Suprit Singh, Maruti Kumaran, Kewal Krishan

**Affiliations:** 1 Department of Radiology, Temple University Hospital, Philadelphia, USA; 2 Department of Cardiovascular Surgery, Temple University Hospital, Philadelphia, USA

**Keywords:** bullet migration, cardiothoracic radiology, cardiothoracic & vascular surgery, bullet emboli, bullet fragment, bullet embolism, trauma, gunshot injury to chest

## Abstract

Bullet embolization is an uncommon event after a penetrating gunshot wound. This phenomenon can occur in the venous or arterial vasculature, and patients can present with various symptoms. Physicians need to be familiar with the indications to suspect bullet embolization in patients with gunshot wounds to avoid crucial complications. We present two cases of venous bullet embolization following traumatic gunshot injuries. We will review the different types of bullet emboli, complications, and management.

## Introduction

Bullet embolization is a rare outcome of a firearm injury, despite the high rate of firearm injuries in the United States. There are approximately 10 cases of bullet embolization per year [1]. Patients can be asymptomatic or present with life-threatening complications, such as endocarditis, valve dysfunction, stroke, sepsis, and ischemia [2]. Treatment options include conservative management, surgical intervention, or endovascular retrieval. We will present two cases of bullet embolism via the venous system to the right lower lobar pulmonary artery and the right ventricle.

## Case presentation

Case 1

**Figure 1:**
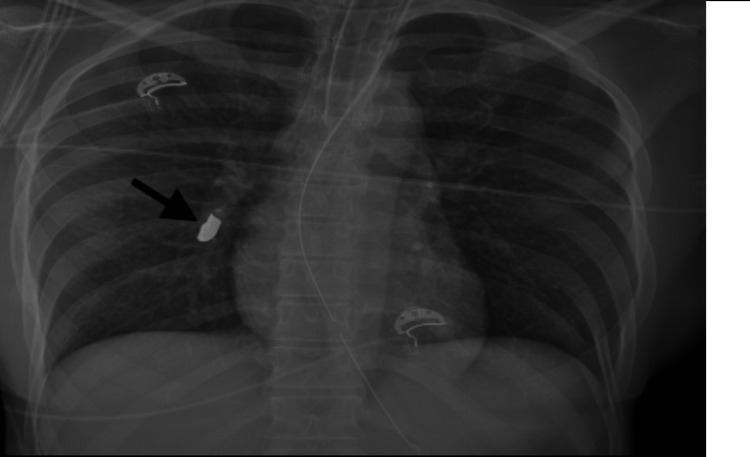
AP chest radiograph: Ballistic fragment projecting over the right lower lung zone. No subcutaneous emphysema or pneumothorax visualized to suggest direct penetrating trauma to the thorax. AP: anterior posterior.

A 32-year-old female with no significant past medical history presented to the emergency department with a Glasgow Coma Scale of 6 after multiple gunshot wounds to the perineum, left lower quadrant of the abdomen, and bilateral lower extremities. In the trauma bay, a chest X-ray showed a ballistic fragment projecting over the right hemithorax without evidence of penetrating injury to the thorax on physical examination or radiograph (Figure 1). The Focused Assessment with Sonography in Trauma (FAST) exam was positive, and the patient was emergently taken to the operating room for exploratory laparotomy for small bowel repair, laceration repair of the liver, complex cystorrhaphy, cholecystectomy, bilateral peri-clitoral, labial, and periurethral laceration repair. Intraoperatively, there was no evidence of diaphragmatic injury.

**Figure 2:**
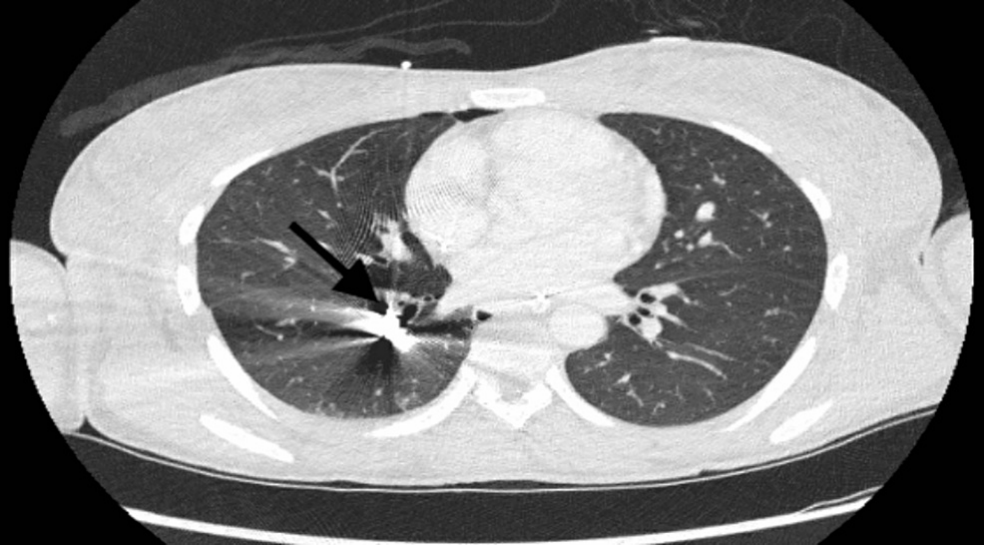
Axial CT chest: streak artifact from bullet within the right lower lobar pulmonary artery. No evidence of pulmonary laceration, contusion, soft tissue injury, or subcutaneous emphysema to suggest direct thoracic injury.

**Figure 3:**
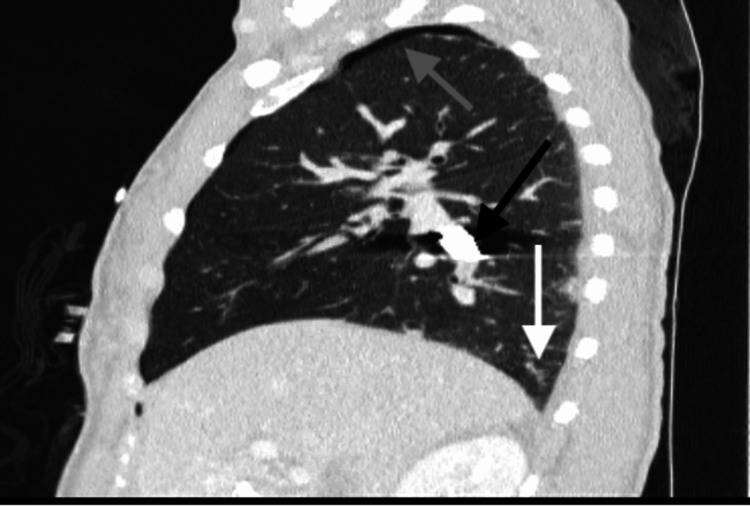
Sagittal CT chest reveals bullet is within the right lower lobar pulmonary artery (black arrow). Small right hemopneumothorax is partially visualized (gray arrow and white arrow).

**Figure 4:**
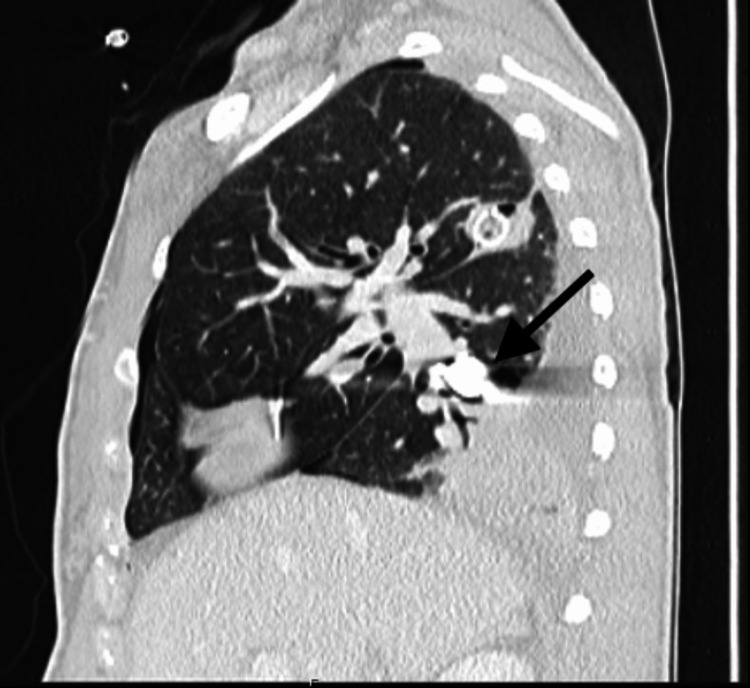
Sagittal CT chest reveals stable position of bullet with subsequent atelectasis in the posterior segment of the right lower lobe due to evolving infarction and collapse. Interval placement of the right apically oriented chest tube due to right hemopneumothorax.

On postoperative day one, a CT chest with contrast showed a ballistic fragment within the right lower lobar pulmonary artery and a small right-sided hemopneumothorax. There was no evidence of pulmonary contusions, lacerations, or penetrating wound tracks through soft tissues to suggest direct injury to the thorax. The additional filling defects in the right lower lobar pulmonary artery suggested decreased flow distal to the obstruction, resulting in right lower lobe pulmonary infarction (Figures 2-3). Concurrent CT abdomen and pelvis revealed a penetrating injury to the perineum, with the projectile tract extending superiorly, likely traversing intra-abdominally, along the lacerated medial right hepatic lobe and entering the hepatic inferior vena cava, where the bullet was lodged into the right lower lobar pulmonary artery. A right apically oriented chest tube was inserted due to the small right hemopneumothorax. On postoperative day 6, a repeat CT chest was performed to assess the embolized bullet and revealed a stable position of the bullet within the right lower lobar pulmonary artery with poor enhancement and subsequent atelectasis of the posterior segment of the right lower lobe due to evolving infarctions (Figure 4). The postoperative course was complicated by pelvic abscesses requiring antibiotics. On postoperative day 22, the patient was discharged from the hospital.

Case 2

**Figure 5:**
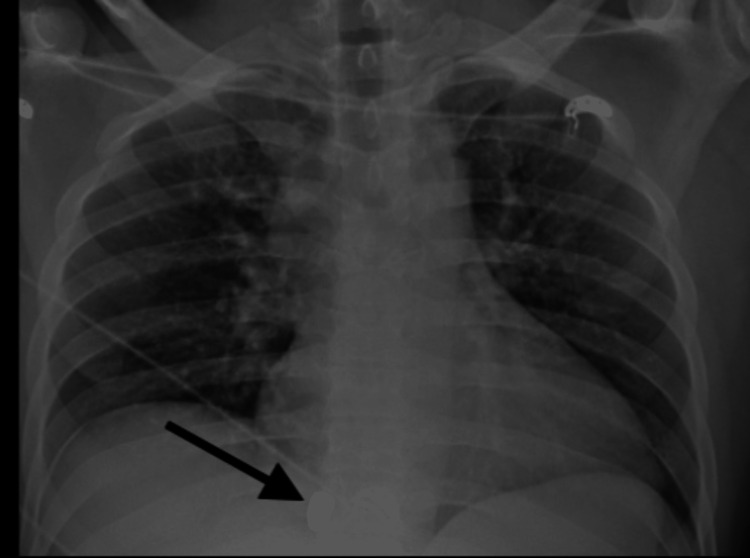
Supine AP chest radiograph demonstrates ballistic fragment projecting over the right heart. AP: anterior posterior.

**Figure 6:**
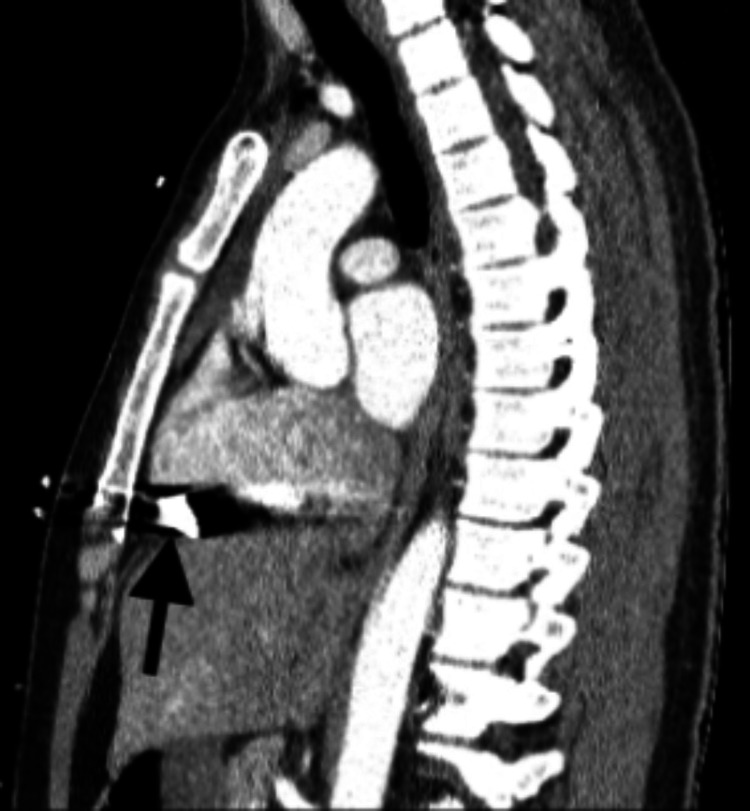
Sagittal CT chest demonstrates bullet lodged within the right ventricle of the heart. No evidence of pneumomediastinum, subcutaneous emphysema, fractures, or pulmonary lacerations to suggest the bullet directly entered the thoracic cavity.

A 29-year-old male with no significant past medical history presented to the emergency department with multiple gunshot wounds to the abdomen, including the liver. In the trauma bay, a chest X-ray showed a retained ballistic projectile overlying the right heart without pneumothorax or hemothorax, with concerns of pericardium injury. He was emergently taken to the operating room for an exploratory laparotomy and pericardial window (Figure 5). Exploratory laparotomy revealed liver and inferior vena cava injuries, presumably from the bullet's trajectory. Intraoperatively, a trans-diaphragmatic pericardial window was negative for visualization of the bullet. On postoperative day 1, the CT chest showed the bullet within the right ventricle, concerning bullet embolization entering from the hepatic IVC to the right ventricle. Cardiothoracic surgery was consulted, and an intraoperative transesophageal echocardiogram (TEE) was performed to visualize the exact position of the bullet. Intraoperative TEE was negative for visualization of the bullet, and an intraoperative chest radiograph demonstrated a persistent bullet projecting over the right heart. The decision was made to perform a median sternotomy, where the bullet was lodged within the right ventricle muscles anteriorly and immediately beneath the tricuspid valve (Figure 6). Given the bullet's position within the right ventricle immediately beneath the tricuspid valve, it was difficult to visualize the TEE and pericardial window.

**Figure 7:**
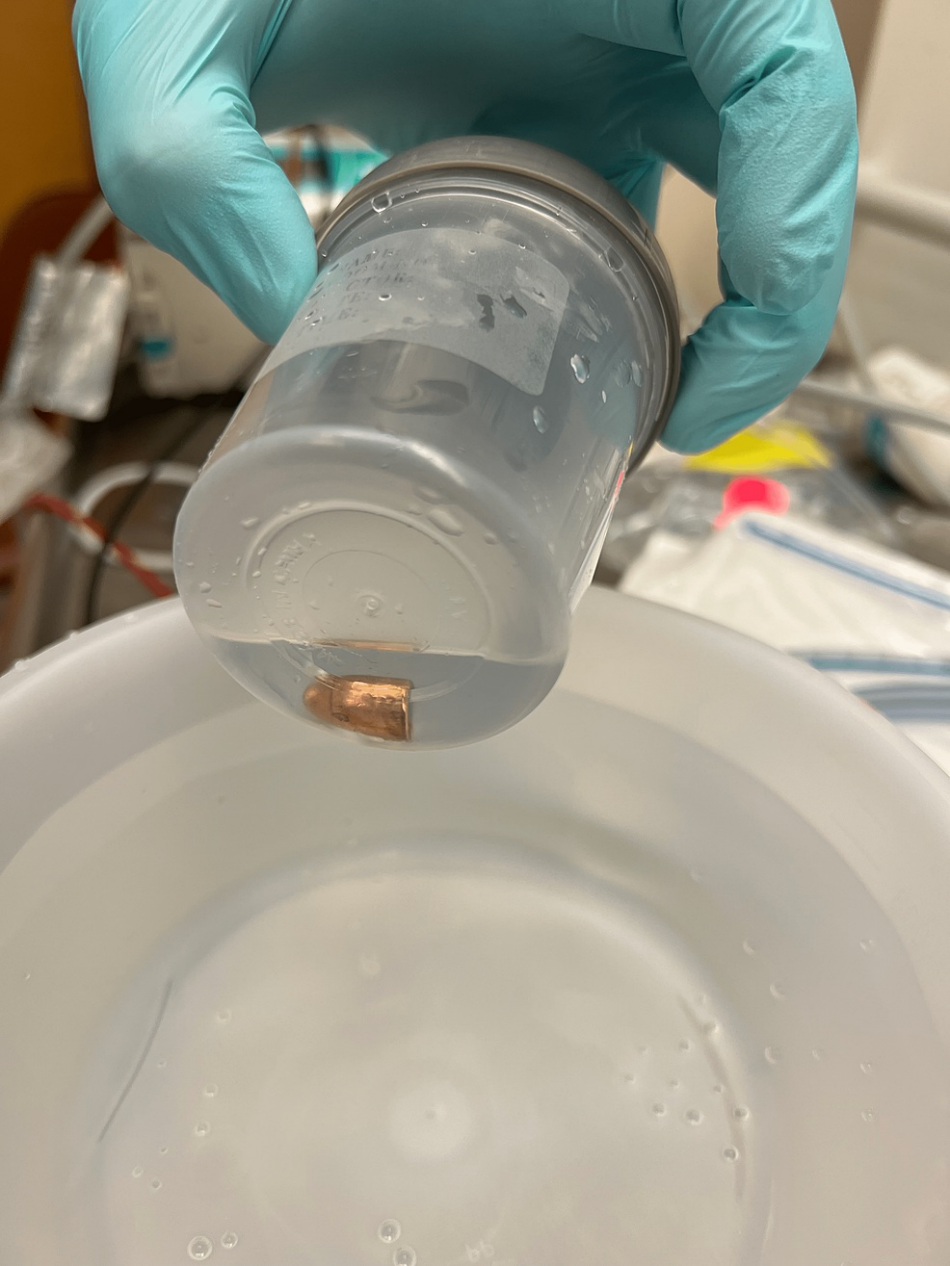
Bullet in specimen cup after removal.

**Figure 8:**
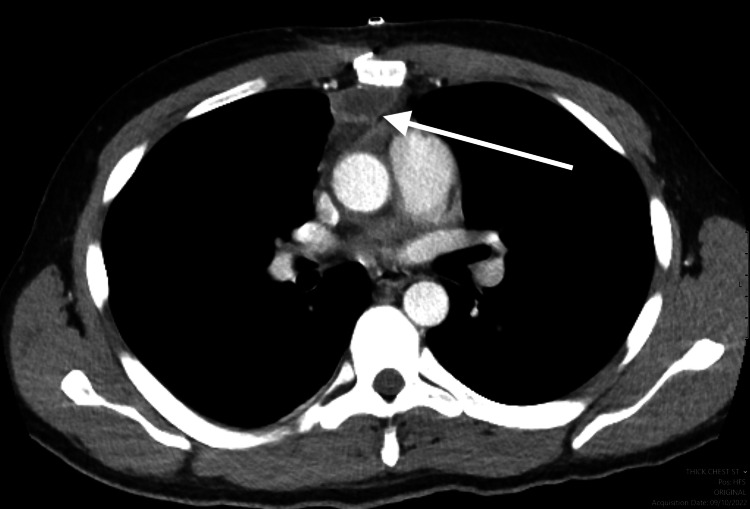
Axial CT chest reveals fluid collection with peripheral rim enhancement in the retrosternal region. Interval removal of bullet from the right ventricle postoperatively.

The bullet was subsequently removed (Figure 7). Since there were no injuries within the thorax and no evidence of diaphragmatic injury intraoperatively, bullet embolization from the hepatic inferior vena cava into the right atrium and subsequently within the anterior muscles of the right ventricle was presumed. Postoperatively, the patient had a complicated course, with an electrocardiogram revealing pericarditis and persistent leukocytosis up to 22,000 cells/mm^3^. Colchicine was administered to treat the pericarditis, and despite compliance with therapy, leukocytosis persisted. On postoperative day 15, a repeat CT chest revealed acute mediastinitis with retrosternal fluid collections extending superiorly into the superior mediastinum (Figure 8). Intravenous antibiotics were started at this time. Interventional radiology was consulted for retrosternal abscess drain placement. On postoperative day 17, procedure images with interventional radiology revealed a decreasing fluid collection, and as a result, there was no safe window to place a drain. The procedure was deferred for continued antibiotic therapy. On postoperative day 21, the patient was discharged home with antibiotics. During outpatient follow-up with trauma surgery on postoperative day 56, the retrosternal abscess resolved, and the patient had no complications at this visit.

## Discussion

Bullet embolization can occur with the bullet entering the arterial or venous system or via deterioration of a vessel lumen due to the throbbing action of the vessel against the missile [3]. Although the majority of embolization occurs in an antegrade pattern by entering the arterial system, 15% of the 153 known bullet embolizations occurred in a retrograde pattern by entering the venous system [4,5]. Paradoxical embolization can occur through a right-to-left shunt, most commonly through a patent foramen ovale, resulting in bullet embolization from the right heart to the left heart, and occurs in five percent of known bullet embolizations [6]. Our cases describe embolization through the venous system, most commonly the inferior vena cava. However, the femoral and subclavian veins are potential access points for the bullet to embolize the heart and pulmonary system [7]. The first case demonstrates lodgment of the bullet within the pulmonary arterial branches of the right lower lobe with subsequent lung infarction due to poor perfusion. The bullet could not be visualized within the right ventricle with TEE due to its position directly under the tricuspid valve and embedded within the anterior muscles of the right ventricle.

Bullet retrieval is selected when the bullet is lodged within the myocardium, where an endovascular approach or open cardiothoracic surgery with or without cardiopulmonary bypass have been viable retrieval options. Open cardiothoracic surgery has been the most common approach when the bullet is embedded in the myocardium or its size is greater than 5 mm [2,7]. Other indications for open cardiothoracic surgery include symptomatic patients or bullets in the left side of the heart [7]. Endovascular retrieval has increased in frequency due to minimally invasive techniques, although open cardiothoracic surgery for bullet retrieval typically occurs when endovascular retrieval is unsuccessful [1,8].

Our first case presented had no attempted retrievals due to the bullet lodged within the right lower pulmonary arterial branch. Given the large size of the bullet, further distal embolization into smaller vessels or embolization into the left heart could not occur. Irreversible pulmonary infarction and subsequent right lower lobar atelectasis had occurred in a relatively short time, and the patient was asymptomatic from bullet embolization into the pulmonary vascularization. The patient was treated with supportive care, and they have not demonstrated any known current complications from retained bullet embolism within the pulmonary vasculature. Another case treated with conservative management was reported by Bakan et al., showcasing a bullet presumably embolized from the right hepatic vein to the heart. The pellet was lodged in the interventricular septum, and the patient underwent multiple unsuccessful endovascular retrievals of the bullet. As a result, the patient was treated with conservative management. Since the patient was asymptomatic and the bullet remained stable over the next few months of follow-up, no cardiothoracic surgery was attempted [2]. However, this case reported by Bakan et al. differs from our first case because they did not initially try conservative management before the endovascular attempt.

Our second case presented used open cardiothoracic surgery for retrieval due to the bullet being partially lodged within the myocardium of the right ventricle. Endovascular retrieval was not utilized due to the inability to visualize the bullet on TEE, and the exact position was challenging to determine despite radiographs and cross-sectional imaging. Due to the potential to embolize distally and potentially injure critical vasculature, open cardiothoracic surgery was deemed appropriate. In a case that de Sousa Arantes Ferreira et al. reported, the patient underwent open surgery because the bullet's size was greater than 5 mm and its location was inside the right ventricle. In our case and the case reported by de Sousa Arantes Ferreira et al. [7], the location of the missile was crucial in the decision to undergo surgical management to prevent the bullet from embolizing further with indeterminate consequences. Lastly, in a case reported by Busada et al., a bullet was located within the intrahepatic IVC, and the patient was treated with a hybrid approach with open and endovascular surgery. The open cardiothoracic surgery with cardiopulmonary bypass was completed first to complete the IVC repair. The patient’s chest was closed, and he was weaned from cardiopulmonary bypass before undergoing inferior vena cavography with vascular surgery and interventional radiology [5]. This case by Busada et al. showcased a hybrid approach to management.

Our second case highlights the complications after open cardiothoracic surgery, including the development of subsequent acute mediastinitis and retrosternal abscesses. Both complications were successfully treated with antibiotic therapy. The complication of acute mediastinitis and retrosternal abscess formation after bullet retrieval is a general complication of open cardiothoracic surgery and is not specific to bullet retrieval.

## Conclusions

Bullet embolization after penetrating injury via the arterial or venous system is a rare outcome of firearm injury but has potentially fatal complications. Our cases demonstrate the necessity of collaboration between the Department of Cardiothoracic Surgery and Radiology and multimodality imaging, including radiographs, CT scans, and echocardiograms, to determine the specific location of the embolized bullet to guide further treatment and possible retrieval.
